# Supplementary material for: Dissecting the Heterogeneous Cortical Anatomy of Autism Spectrum Disorder Using Normative Models
Source: Biol Psychiatry Cogn Neurosci Neuroimaging. 2019 Jun;4(6):567–78. doi: 10.1016/j.bpsc.2018.11.013 (PMC6551348; doi:10.1016/j.bpsc.2018.11.013)
Supplement: Supplemental Material [file mmc1.pdf]

Dissecting the Heterogeneous Cortical Anatomy of Autism Spectrum Disorder Using Normative Models

Supplementary Information

Supplementary methods

Exclusion criteria

Exclusion criteria for the Longitudinal European Autism Project (LEAP) study included a history of substance abuse and standard neuroimaging contraindications (e.g. claustrophobia, metal implants). Individuals were also excluded if they had a history of bipolar disorder or psychosis. In contrast to case-control studies that aim to detect consistent group differences, here we were interested in characterizing the heterogeneity within autism spectrum disorder (ASD) at the level of the individual. Therefore, we did not exclude other comorbidities in the clinical group because up to 70% of ASD individuals have one or more psychiatric conditions (1) and 30-50% of individuals with ASD are on stable medications (2).

Magnetic Resonance Imaging

A high resolution T1-weighted image was acquired from each participant with a standard Alzheimer's Disease Neuroimaging Initiative (ADNI) sequence (3), matched across scanning sites. Cortical thickness was estimated from the high-resolution T1-weighted image for each subject using FreeSurfer version 5.3 (http://surfer.nmr.mgh.harvard.edu/). Prior to analysis, all surface reconstructions were visually assessed for reconstruction errors by at least 3 independent raters. We excluded a small number of scans with severe artifacts (e.g. caused by head motion). The rest of the scans were included 'as is', i.e. we did not allow manual edits to reduce the possibility of bias (e.g. due to individual differences in operator skill). Cortical thickness maps were then smoothed with a 10-mm surface-based Gaussian kernel.

Gaussian process regression

As mentioned in the main text, Gaussian process regression (GPR) (4) was used to estimate separate normative models of cortical thickness (CT) and surface area (SA) at each vertex on the cortical surface. Whilst other methods are also suited to this purpose (e.g. Bayesian polynomial regression), in preliminary testing we found that GPR provides superior estimation of the mean and the ability to map the variation across the cohort through centiles of predictive confidence. We refer the reader elsewhere for a full treatment of Gaussian processes (5, 6) but briefly, a Gaussian process (GP) specifies a distribution over

functions, such that any finite number of elements has a joint Gaussian distribution. They are excellent tools for Bayesian regression: given a dataset specified by $\mathcal{D} = \{\mathbf{x}_i, y_i \in\}_{i=1}^{N}$ – where $\mathbf{x}_i$ are D-dimensional vectors of covariates, N is the total sample size and $y_i \in \mathbb{R}$ are response variables – the response variables are predicted using a potentially nonlinear regression model with additive Gaussian noise, i.e.: $y_i = f_i + \epsilon_i$ where $\epsilon_i \sim N(0, \sigma_n^2)$. Inference then proceeds by placing a GP prior over this function then computing the posterior distribution using the canonical GPR predictive equations (5). This prior is uniquely specified by a mean ($m(x)$) and covariance ($k(x, x')$) function. Here, without loss of generality we choose a mean function equal to zero and a generic covariance function combining linear and non-linear terms, i.e.:

$$k(\mathbf{x}_i, \mathbf{x}_j) = \mathbf{x}_i^T \mathbf{x}_j + \sigma_f \exp\left(-\frac{1}{2}(\mathbf{x}_i - \mathbf{x}_j)^T \mathbf{\Lambda}(\mathbf{x}_i - \mathbf{x}_j)\right)$$

Where σ_f is a signal amplitude parameter for the nonlinear component and $\mathbf{\Lambda}$ is a diagonal matrix with ℓ_d^{-2} along the leading diagonal. These are 'automatic relevance determination' parameters (5) that can down-weight irrelevant dimensions in the input space or emphasize important dimensions. Training a GP model refers to finding the optimal values for the model parameters which are: $\ell_1, \ldots, \ell_D, \sigma_n$ and σ_f. This is conveniently achieved by maximizing the logarithm of the model evidence (i.e. the denominator of Bayes rule). Finally, we compute a single subject Z-statistic image for each subject (i) and at each brain location (j) by computing:

$$z_{ij} = \frac{y_{ij} - \hat{y}_{ij}}{\sqrt{\sigma_{ij}^2 + \sigma_{nj}^2}}$$

Here, $\hat{y}_{ij}$ is the predicted mean and predicted variance, σ_{ij}, which is combined with the true response (y_{ij}) and variance learned from the TD distribution (σ_{nj}). Because we estimate a separate noise parameter for each vertex, this should accommodate regional differences in population variation (for example, the estimated variance parameter will be higher in the regions where there is greater variation across individuals).

Cross-validation

To assess generalization, we used 10-fold cross-validation where we partitioned the data into 10 'folds' and repeatedly trained the model on 90% of the data, withholding the remaining 10% for estimating generalization performance. This was repeated 10 times so that each partition was excluded once. This

procedure is standard in machine learning and is known to provide approximately unbiased estimates of the true generalization ability.

Post-hoc investigation of potential confounding variables

To investigate the potential confounding effect of various potential confounding variables, we performed several tests. As described in the main text, we first estimated a normative model for CT additionally including scanning site, IQ and the FreeSurfer Euler number (EN) (7) as covariates. In addition, we additionally performed several post-hoc tests for potential confounding variables including scan quality, IQ and comorbid attention deficit/hyperactivity disorder (ADHD) symptoms. However, we emphasize strongly that these should be considered as illustrative only, because our study design does not allow us to determine the direction of cause-effect relationships. For example, it is reasonable to expect that subjects that show the most atypical cortical anatomy may also express the highest level of symptoms, have the most intellectual impairment and be the most likely to suffer from comorbid symptoms. First, to assess the possibility of scan quality (e.g. due to excessive head motion in the scanner) influencing our results, we correlated (using Spearman correlation) the deviations from the model with the Euler number. The EN summarizes the topological complexity of the estimated cortical surface and has been proposed as a proxy measure of scan quality (8) . However, this is an indirect measure in that it does not model scan quality directly, it should be considered with caution since many other variables can potentially influence EN, including age, atypicalities in cortical anatomy and disorder severity. Therefore, we also correlated EN with age and with ASD symptoms. In addition, we correlated the deviations from the normative with measures of full-scale IQ (see (9)) and with measures of comorbid ADHD symptoms derived from the Development and Well-being Assessment (10). We refer the reader elsewhere for a detailed description of these measures (3, 9).

Supplementary results

Age histogram

Figure S1 shows the distribution of subject ages across diagnoses.

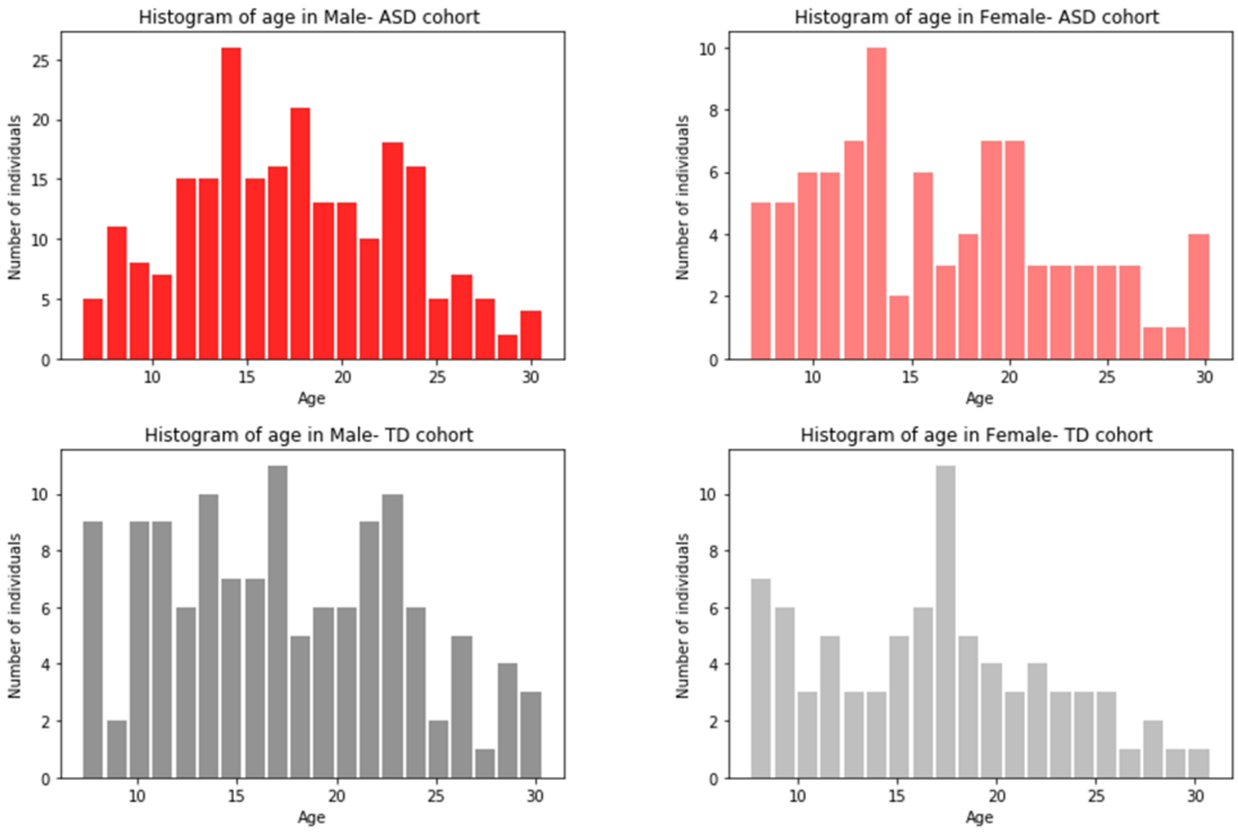

Supplementary Figure S1: Histogram of age of Female and Male individuals across TD and ASD cohort.

Model fit evaluation

Figure S2 shows the mean accuracy of the normative model for predicting CT in typically developing (TD) and ASD participants, both in terms of root mean squared error (Figure S2A) and correlation between true and predicted CT values (Figure S2B).

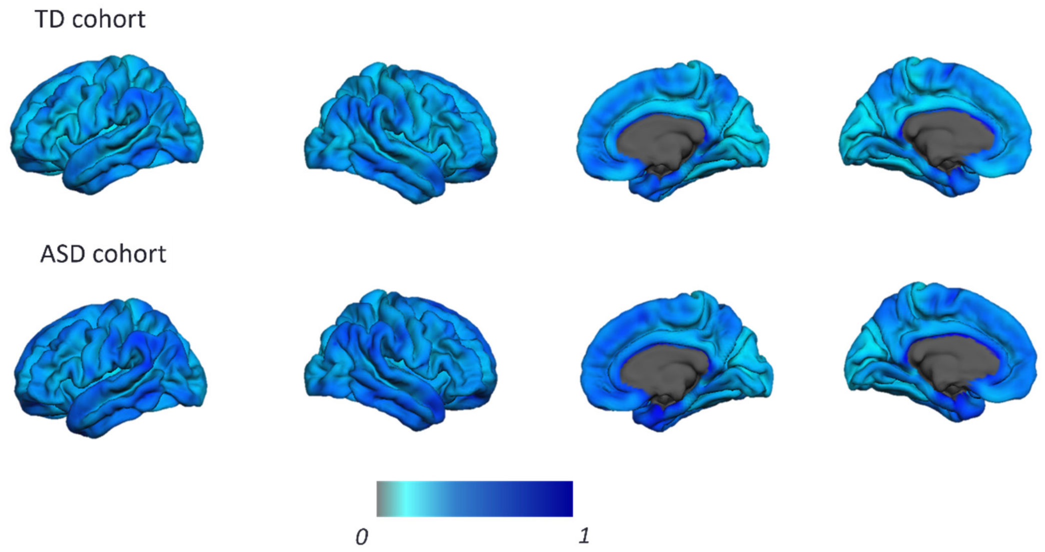

Supplementary Figure S2.A: Root mean square error of true and prective mean of cortical thickness in TD cohort.

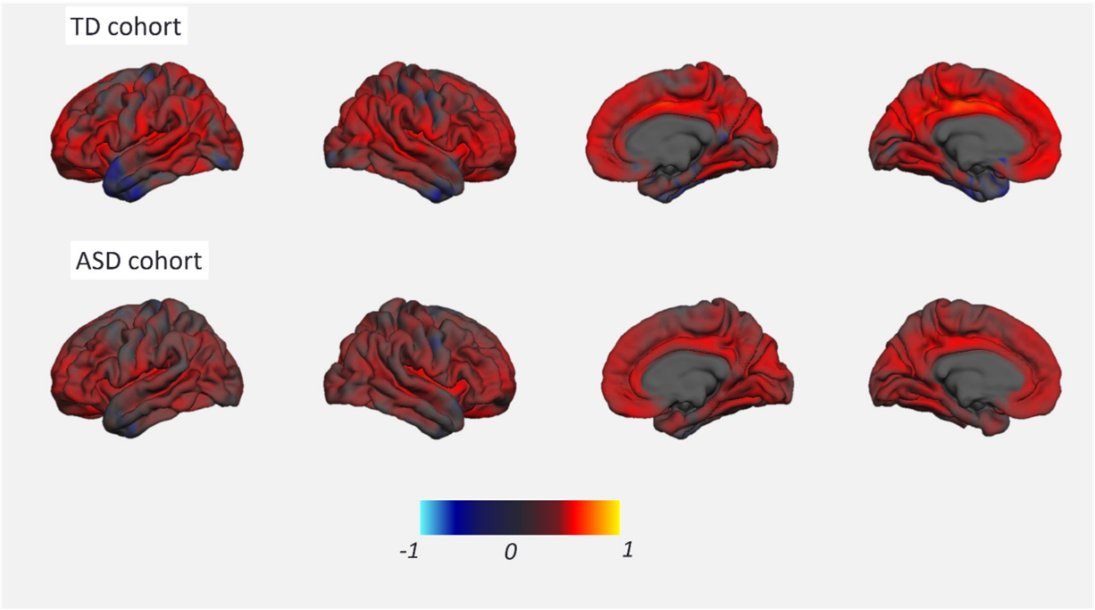

Supplementary Figure S2.B: The correlation between true and prective mean of cortical thickness in TD and ASD cohort.

Normative developmental changes for cortical thickness in females

Supplementary Figure S3 shows the predictions made by the normative model for changes in female TD subjects (see Figure 2 in the main text for males).

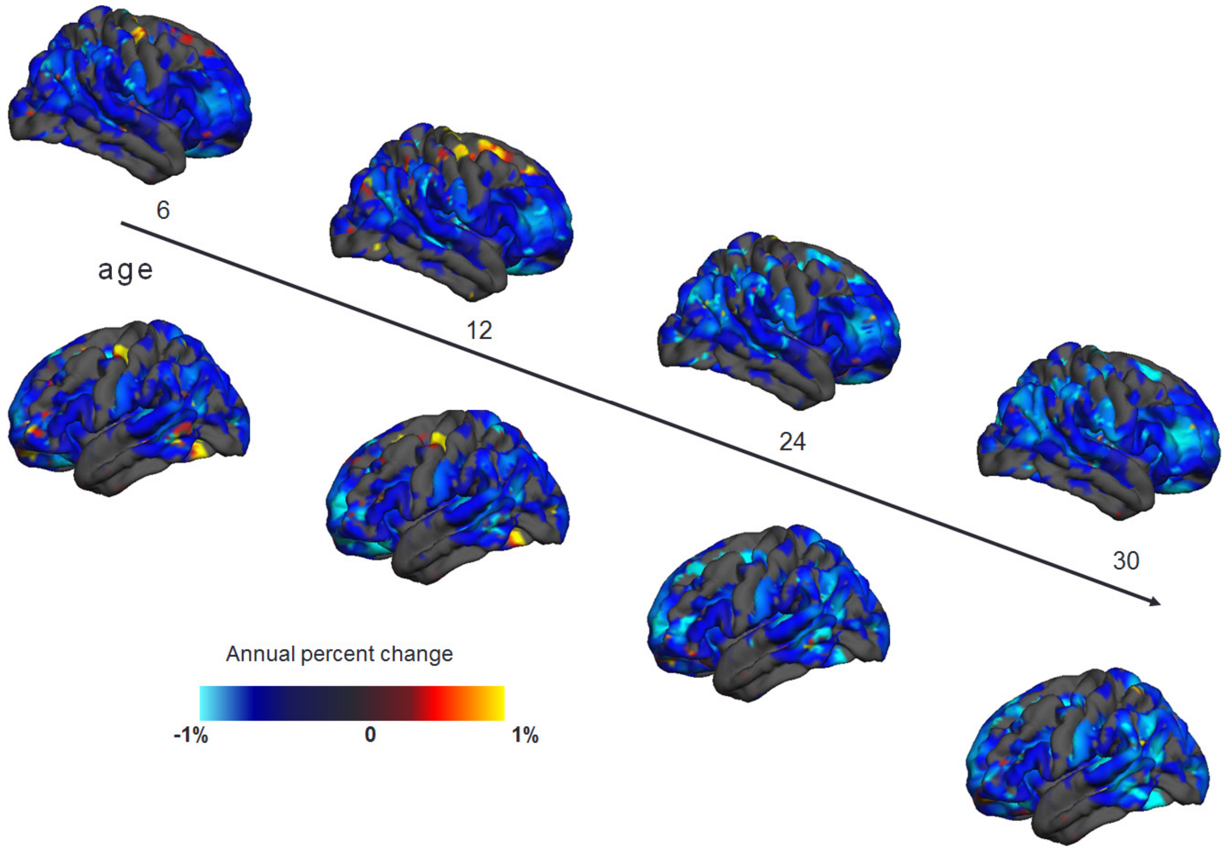

Supplementary Figure S3: Normative model of developmental changes of cortical thickness across the developmental range in the typical developing female cohort. Cortical thickness was predicted using a trained normative model across the age range of six to thirty-one. The predicted cortical thickness map was thresholded so that only vertices that could accurately predict the true cortical thickness in the healthy cohort under cross-validation were retained (Pearson correlation, $p < 0.05$, FDR). Blue vertices and yellow indicate reduced and increased CT respectively.

Normative model of cortical thickness using age, gender, site, IQ and FreeSurfer Euler number as covariates

Supplementary Figures S4 and S5 show deviations from the normative model for CT re-estimated after additionally including IQ addition and scanning site dummy variables and FreeSurfer Euler number as covariates, separately for positive (Figure S4) and negative (Figure S5) deviations. The differences between this model and the original model are negligible and all the conclusions remain unchanged.

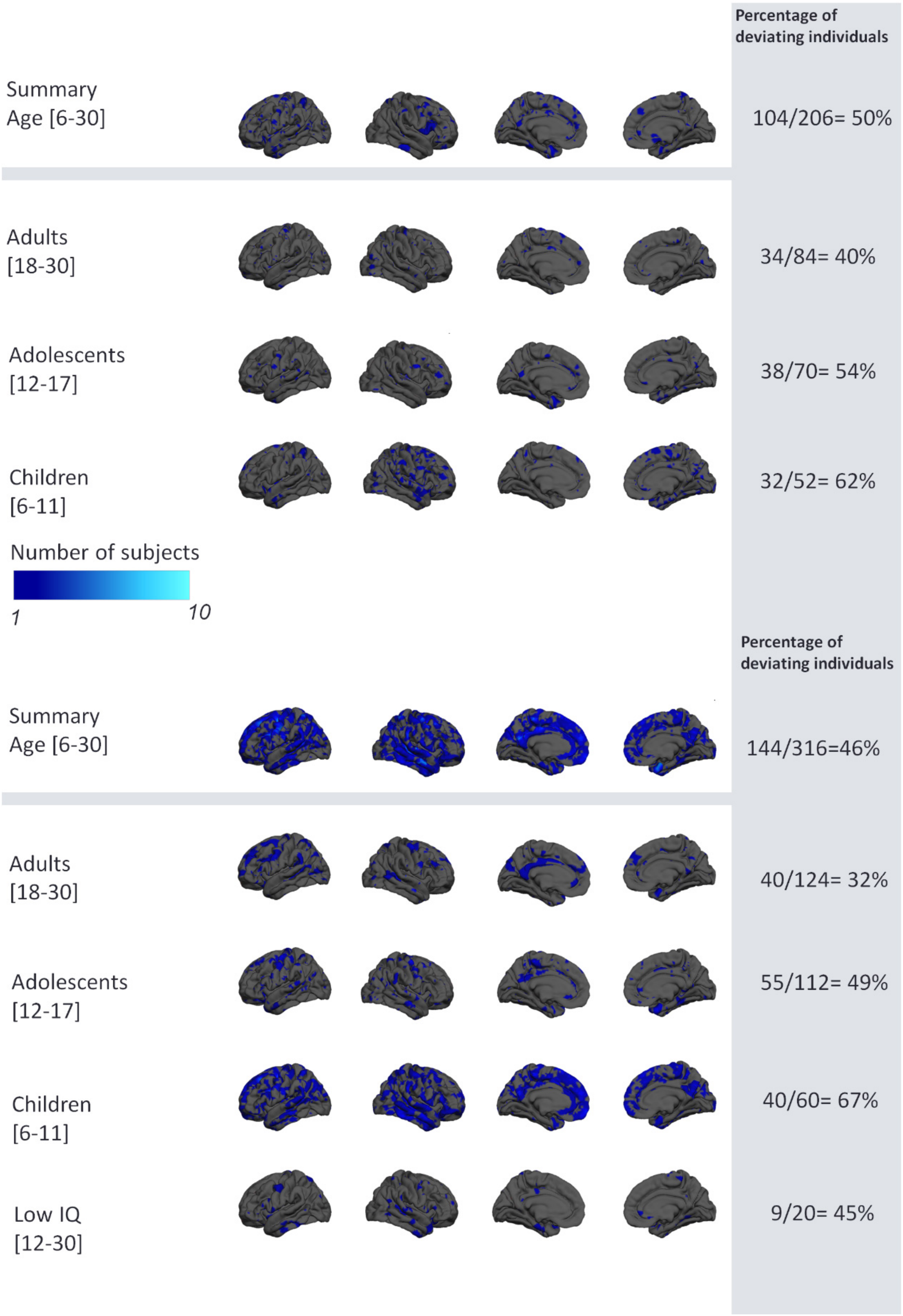

Supplementary Figure S4: Overlap of vertex-wise negative deviation across each cohort and schedule. This map shows the number of subjects with significant deviations in each vertex after FDR correction.

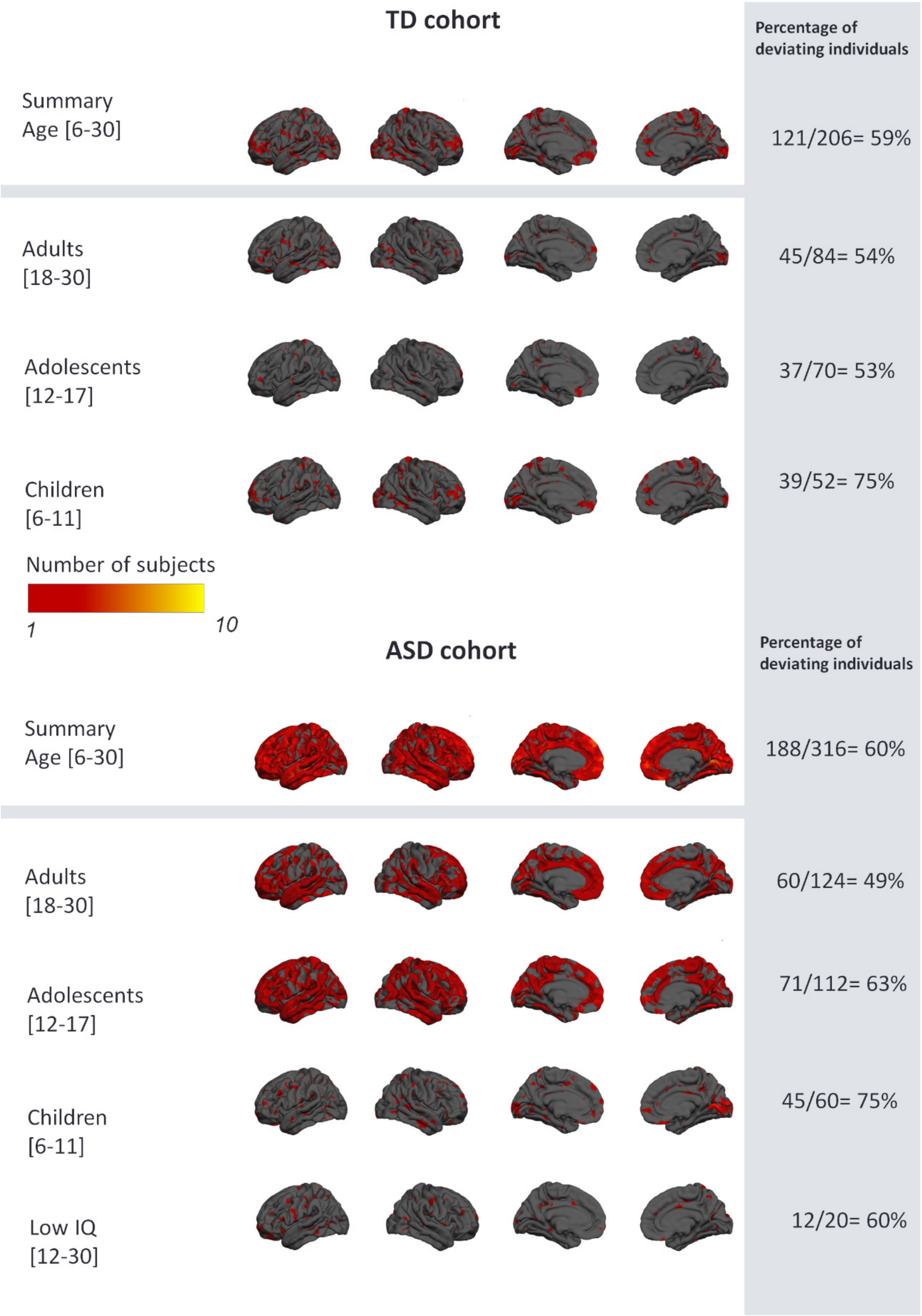

Supplementary Figure S5: Overlap of vertex wise positive deviation across each cohort and schedule.

Normative model of surface area

Supplementary Figure S6 and Figure S7 show deviations from the normative model for CT estimated using age and gender as covariates, separately for positive (Figure S6) and negative (Figure S7) deviations. The overlap of deviating voxels shows a similar but slightly different pattern relative to CT.

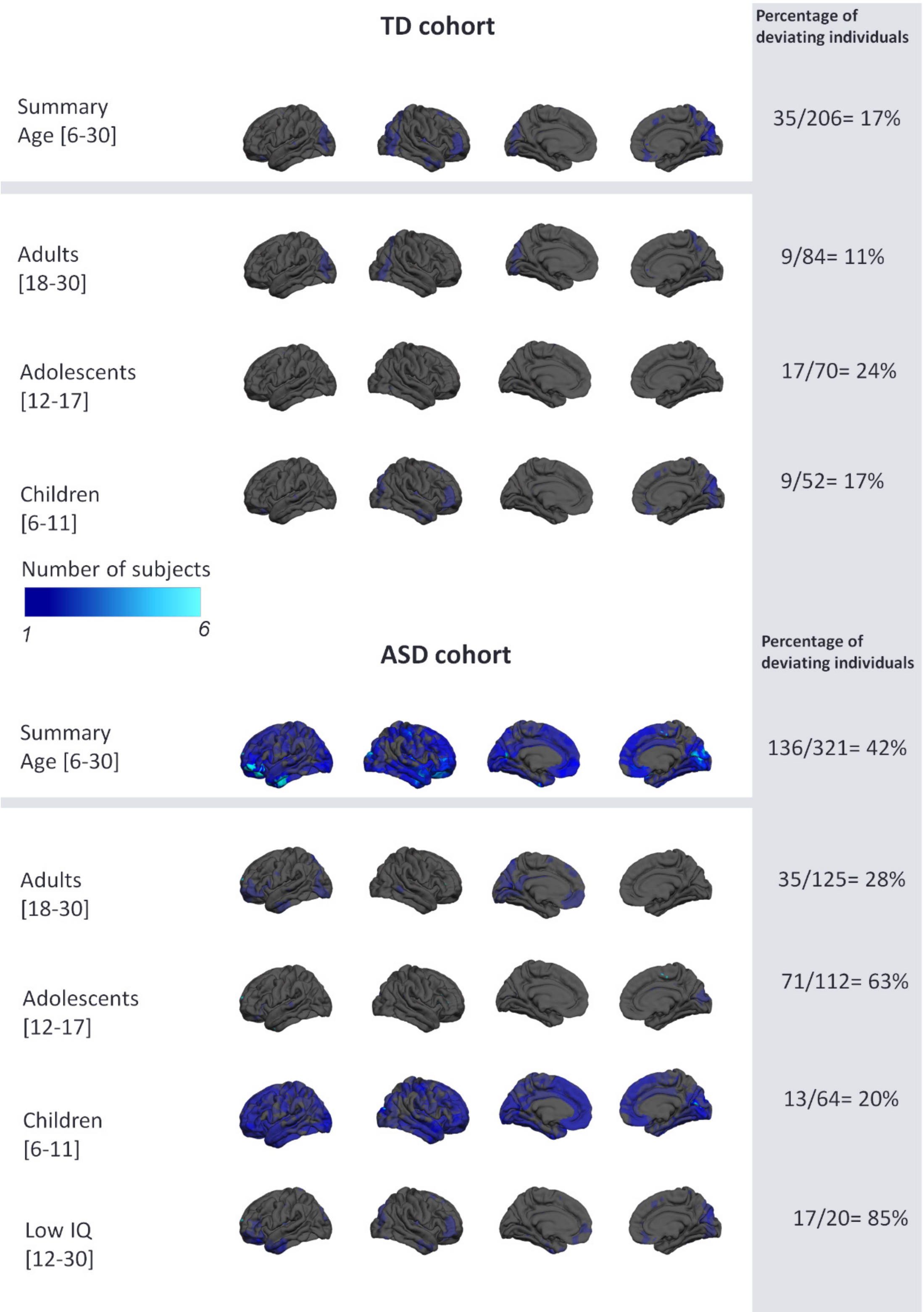

Supplementary Figure S6: Overlap of vertex-wise negative deviation in surface area normative model across each cohort and schedule. This map shows the number of subjects with significant deviations in each vertex after FDR correction.

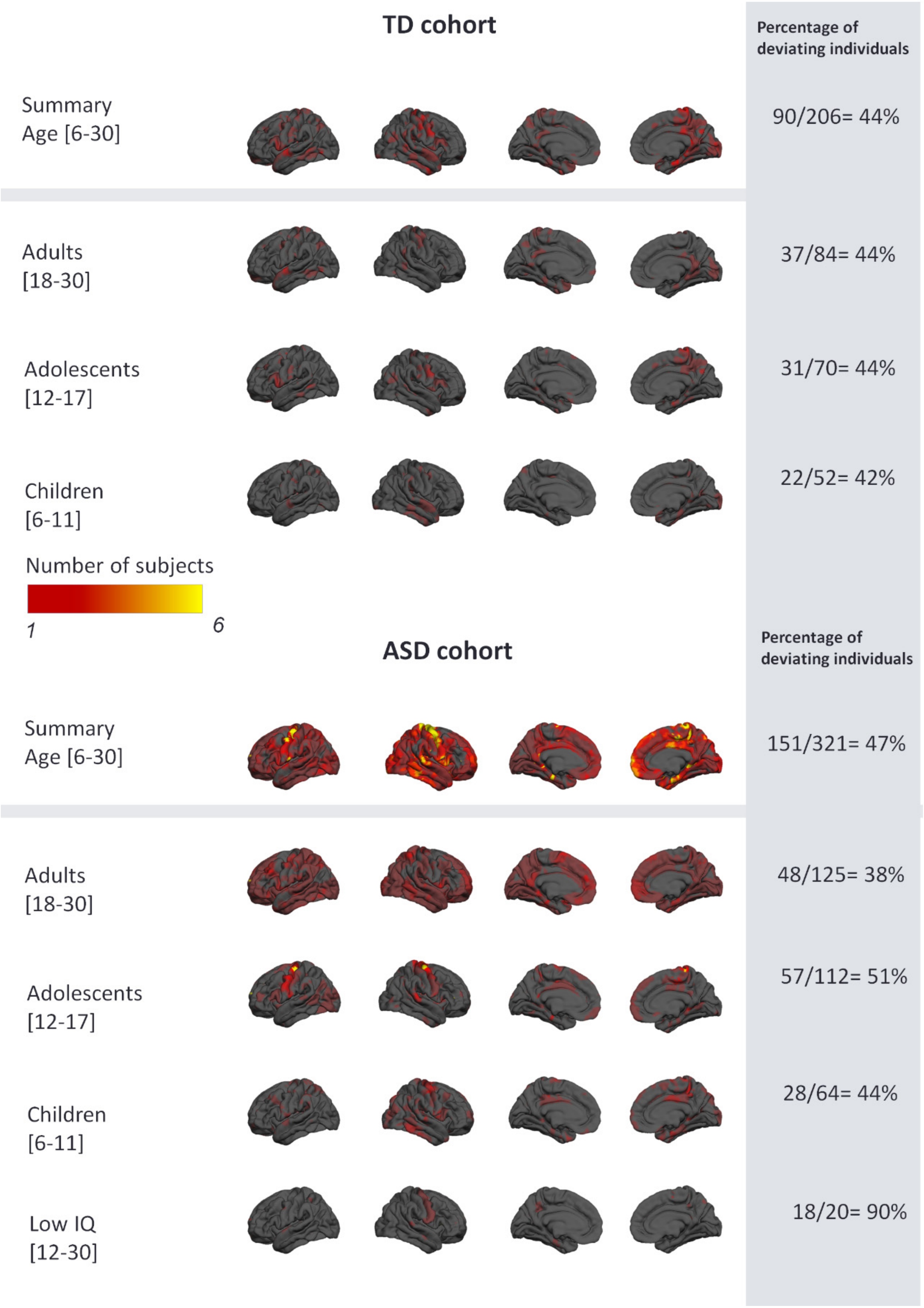

Supplementary Figure S7: Overlap of vertex wise positive deviation across each cohort and schedule.

Individual subject deviations

Supplementary Figure S8 shows the top 15 subjects deviating from the normative pattern for CT.

EV rank	Age	Sex
14	6.45	M
10	8.38	M
1	9.69	M
15	9.79	M
6	13.60	M
4	15.24	M
9	15.38	M
12	16.25	M
8	18.24	M
2	22.58	M
5	6.24	F
11	7.88	F
13	10.19	F
7	10.68	F
3	18.66	F

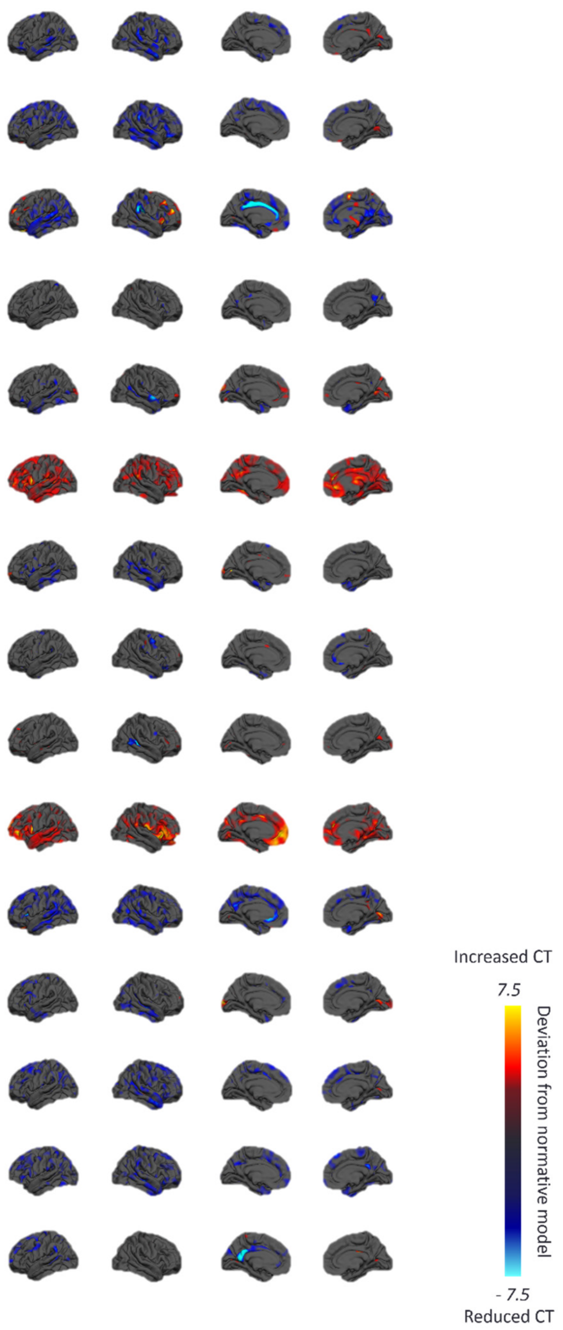

Supplementary Figure S8: NMPs of top fifteen deviating individuals from normative CT model. These subjects who belong to ASD cohort have highly individualized patterns of deviation with respect to brain regions and different sign of the deviation.

Individual subject deviations

Supplementary Table S1 shows the clinical characteristics of the sample separately for each site.

Supplementary Table S1: Clinical characteristics for each site.

	Cambridge		KCL		Mannheim		Nijmegen		Rome		Utrecht	
**Variable**	***ASD***	***TD***	***ASD***	***TD***	***ASD***	***TD***	***ASD***	***TD***	***ASD***	***TD***	***ASD***	***TD***
Age, mean, [SD]	17.6 [5.8]	17.0 [6.4]	16.9 [5.9]	18.7 [6.6]	15.4 [3.4]	15.3 [3.6]	16.2 [5.6]	15.0 [4.1]	24.9 [2.9]	24.9 [3.5]	16.8 [5.5]	16.7 [6.2]
IQ, mean [SD]												
Global IQ	106 [19]	114 [11]	100 [21]	111 [16]	102 [13]	109 [15]	97 [18]	101 [14]	101 [14]	106 [10]	105 [13]	111 [8]
Performance IQ	109 [21]	116 [12]	100 [20]	110 [16]	104 [15]	113 [13]	97 [22]	102 [18]	104 [18]	103 [15]	107 [17]	109 [12]
Verbal IQ	103 [17]	109 [11]	99 [20]	111 [18]	101 [15]	101 [14]	97 [19]	100 [15]	98 [16]	109 [8]	105 [14]	113 [14]
ADI-R [SD]												
Social	17.2 [6.6]	-	17.9 [6.4]		15.2 [7.2]	-	14.4 [6.4]	-	11.4 [5.7]	-	16.3 [5.8]	-
Communication	14.5 [2.7]	-	15.2 [5.4]	-	10.4 [4.9]	-	12.7 [5.4]	-	9.3 [5.4]	-	11.3 [5.4]	-
Repetitive Behavior	5.0 [2.7]	-	5.0 [2.4]	-	5.1 [3.8]	-	2.9 [2.1]	-	5.3 [2.3]	-	3.5 [2.7]	-
ADOS [SD]												
Total	5.2 [2.4]	-	5.1 [2.9]	-	-	-	5.3 [2.7]	-	-	-	4.8 [2.7]	-
Social	6.3 [1.8]	-	5.3 [2.8]	-	-	-	6.1 [2.5]	-	-	-	5.5 [2.7]	-
Repetitive Behavior	4.5[2.5]	-	5.7 [2.6]	-	-	-	3.7[2.7]	-	-	-	4.3[2.4]	-
Schedule												
A: Adults	16	9	50	33	5	5	23	10	17	10	14	10
B: Adolescents	15	6	34	18	20	11	31	27	0	0	12	8
C. Children	7	7	26	9	3	5	20	18	0	0	8	13
D. IQ < 70	2	-	12	-	0	-	6	-	0	-	0	-

Scan quality associations with extreme deviations and age

In Supplementary Table S2, we show the correlation of deviations with the FreeSurfer Euler number. Note that smaller EN values are indirectly associated with a lower scan quality.

Supplementary Table S2: Correlation between extreme deviations and Euler number.

	Euler number in ASD cohort	Euler number in TD cohort	Euler number overall
**Extreme deviations**	-0.57 *	-0.62 *	-0.58 *
**Age**	0.39 *	0.38 *	0.38 *
**ADI Social**	-0.18 *	-	-
**ADI Communication**	-0.25 *	-	-
**ADI RRB**	-0.25 *	-	-
**ADOS Total**	-0.11	-	-
**ADOS Social**	-0.02	-	-
**ADOS RRB**	-0.21 *	-	-

This shows that EN was correlated with the deviations in both ASD and TD cohorts, all ADI symptom domains, and ADOS repetitive behaviors. Regarding alternative potentially confounding variables, the extreme value deviations were weakly negatively correlated with IQ in the ASD cohort (ρ = -0.16, $p<0.05$), but not in the TD cohort (ρ = 0.08, n/s). They were also correlated with some ADHD symptom scales (Supplementary Table S3).

Supplementary Table S3: Correlation between extreme deviations and ADHD score.

	**ADHD Inattentive parent in ASD**	**ADHD Inattentive parent in TD**	**ADHD Inattentive**
Extreme deviations	0.20 *	-0.01	0.22 *
	**ADHD Hyperimpulsive parent in ASD**	**ADHD Hyperimpulsive parent in TD**	**ADHD Hyperimpulsive**
Extreme deviations	0.24 *	0.16	0.26 *

Taken together these results reinforce the cautions noted above, and preclude a definitive assessment of the degree to which any one particular confounding variable may have influenced our results.

Supplementary Table S4 shows the clinical characteristics from the top deviating subjects from the normative model for CT, all of whom have ASD

Supplementary Table S4: Clinical characteristics of the top fifteen deviating participants from normative CT model. These subjects who belong to ASD cohort, have highly individualized patterns of deviation with respect to brain regions and different sign of the deviation.

		Schedule	Site	Sex	VIQ	PIQ	FSIQ	Age	ADI-social	ADI-communication	ADI-RRB	ADOS-TOTAL	ADOS-SA	ADOS-RRB
**1**	ASD	Children	Mannheim	M	-	69	-	9.69	20	10	8	-	-	-
**2**	ASD	Adults	Rome	M	70	79	76	22.58	18	11	3	-	-	-
**3**	ASD	Adults	Nijmegen	F	79	66	74	18.66	4	6	0	3	4	6
**4**	ASD	Adolescent	Utrecht	M	86	106	96	15.24	10	9	2	5	6	6
**5**	ASD	Children	KCL	F	90	109	100	6.24	17	17	7	10	10	8
**6**	ASD	Adolescent	Nijmegen	M	114	124	118	13.6	15	15	2	-	-	-
**7**	ASD	Children	KCL	F	116	133	127	10.68	10	9	8	1	2	6
**8**	ASD	Adults	KCL	M	87	86	84	18.42	-	-	-	2	2	6
**9**	ASD	IQ < 70	KCL	M	58	59	56	15.83	22	21	9	10	10	10
**10**	ASD	Children	KCL	M	111	106	110	8.38	16	18	5	6	3	9
**11**	ASD	Children	Utrecht	F	93	98	95	7.88	23	17	3	7	5	9
**12**	ASD	IQ < 70	Nijmegen	M	52	50	54	16.25	27	21	6	10	10	8
**13**	ASD	Children	KCL	F	-	-	-	10.19	0	8	2	6	7	6
**14**	ASD	Children	KCL	M	99	107	104	6.45	14	12	5	2	3	1
**15**	ASD	Children	Cambridge	'M	106	128	119	9.79	16	15	2	4	5	6

Supplementary Table S5 shows the clinical characteristics from the top deviating subjects from the normative model for SA.

Supplementary Table S5: Clinical characteristics of the top fifteen deviating participants from normative surface area model. While 80% of the individuals belong to ASD cohort with highly heterogeneous profiles, there are several individuals in the list who belong to TD cohort.

		**Schedule**	**Site**	**Sex**	**VIQ**	**PIQ**	**FSIQ**	**Age**	**ADI-social**	**ADI-communication**	**ADI-RRB**	**ADOS-TOTAL**	**ADOS-SA**	**ADOS-RRB**
**1**	ASD	Adults	Nijmegen	F	71	66	70	17.49	9	14	2	7	8	1
**2**	ASD	IQ <70	Cambridge	M	73	66	67	24.29	10	9	5	9	8	10
**3**	TD	Adults	KCL	M	142	136	142	23.08	-	-	-	-	-	-
**4**	ASD	Adolescents	Nijmegen	M	89	64	78	12.07	17	12	1	3	5	1
**5**	TD	Adults	Cambridge	F	104	117	111	18.26	-	-	-	-	-	-
**6**	ASD	Adolescents	Cambridge	M	103	120	113	14.53	16	12	1	7	8	5
**7**	ASD	Adolescents	KCL	M	144	134	142	16.82	20	12	5	6	6	7
**8**	ASD	Children	KCL	F	98	106	102	9.31	25	20	8	8	7	9
**9**	ASD	Children	KCL	F	116	133	127	10.68	10	9	8	1	2	6
**10**	TD	Adults	Utrecht	M	105	85	96	22.06	-	-	-	-	-	-
**11**	TD	Children	Cambridge	M	108	129	120	8.62	-	-	-	-	-	-
**12**	ASD	Children	Mannheim	M	-	69	-	9.69	20	10	8	-	-	-
**13**	ASD	Adults	KCL	M	133	120	130	19.44	18	19	8	-	-	-
**14**	ASD	Adolescents	Cambridge	F	112	104	109	12.11	27	17	8	2	4	1
**15**	ASD	Children	Nijmegen	M	92	94	93	11.57	24	20	7	1	3	1

Finally, Supplementary Tables S6 and S7 shows the associations for the deviations from the normative model and symptom scales.

*Supplementary Table S6: Clinical relevance of the deviations; Significant correlation (Spearman) between the mean of extreme deviation in each cortical parcel and symptoms measured by ADOS and ADI scores (P_value < 0.05). * indicates the regions survived after FDR correction*

Parcel	Correlation Coefficient, r	Parcel	Correlation Coefficient, r
ADI_social, Female		***ADI_RRB, Male***	
superiortemporal	0.22(L)	superiorfrontal	0.23*(L), 0.2(R)
lateralorbitofrontal	0.22(R)	superiortemporal	0.16(L)
parsopercularis	0.22(R)	insula	0.16(L)
precuneus	0.24(R)	caudalanteriorcingulate	0.2(R)
temporalpole	0.26(R)	fusiform	0.15(R)
ADI_social, Male		paracentral	0.15(R)
superiortemporal	0.17(L)	parstriangularis	0.17(R)
lateralorbitofrontal	0.15(R)	temporalpole	0.16(R)
ADI_communication, Female		***ADOS_II_CSS, Female***	
caudalmiddlefrontal	0.21(L)	parsopercularis	0.25(L)
lateralorbitofrontal	0.25(L)	***ADOS_II_CSS, Male***	
parsopercularis	0.22(L)	entorhinal	0.18(R)
pericalcarine	0.23(L)	medialorbitofrontal	0.19(R)
postcentral	0.22(L)	temporalpole	0.16(R)
precentral	0.23(L)	***ADOS_II_Social Affect, Female***	
superiorparietal	0.23(L)	entorhinal	-0.23(L)
inferiortemporal	0.23(R)	lateraloccipital	-0.25(L)
lateraloccipital	0.30(R)	lingual	-0.22(L)
middletemporal	0.30(R)	middletemporal	-0.24(L)
precuneus	0.21(R)	parsopercularis	0.23(L)
rostralmiddlefrontal	0.21(R)	***ADOS_II_Social Affect, Male***	
ADI_communication, Male		medialorbitofrontal	0.18(R)
superiortemporal	0.17(L)	temporalpole	0.15(R)
supramarginal	0.15(L)	***ADOS_II_RRB, Female***	
insula	0.17(L)	caudalmiddlefrontal	0.33*(L), 0.31(R)
superiorparietal	0.15(R)	entorhinal	0.23(L)
paracentral	0.15(R)	fusiform	0.33*(L)
ADI_RRB, Female		inferiorparietal	0.26(L),0.25(R)
caudalmiddlefrontal	0.31*(L), 0.24(R)	inferiortemporal	0.25(L)
entorhinal	0.24(L)	lateraloccipital	0.23(L)
inferiortemporal	0.22(L), 0.28(R)	lingual	0.38*(L),0.34(R)
lateraloccipital	0.30*(L)	rostralmiddlefrontal	0.23(L),0.31(R)

Parcel	Correlation Coefficient, r	Parcel	Correlation Coefficient, r
lateralorbitofrontal	0.32*(L)	superiorfrontal	0.33*(L),0.24(R)
medialorbitofrontal	0.26(L), 0.24(R)	superiorparietal	0.25(L)
parsopercularis	0.28(L)	supramarginal	0.33(L)
parstriangularis	0.31*(L)	caudalanteriorcingulate	0.26(R)
precentral	0.25(L), 0.25(R)	middletemporal	0.24(R)
precuneus	0.22(L)	paracentral	0.25(R)
superiorparietal	0.24(L)	pericalcarine	0.26(R)
inferiorparietal	0.24(R)	precentral	0.29(R)
middletemporal	0.32*(R)	precuneus	0.29(R)
rostralmiddlefrontal	0.28(R)	frontalpole	0.32(R)
superiortemporal	0.23(R)	***ADOS_II_RRB, Male***	
supramarginal	0.36(R)	lateralorbitofrontal	0.18(L)
ADI_RRB, Male		medialorbitofrontal	0.21(L)
entorhinal	0.19(L)	rostralmiddlefrontal	0.16(L)
inferiortemporal	0.14(L), 0.17(R)	insula	0.16(L)
lingual	0.14(L)	entorhinal	0.15(R)
middletemporal	0.18(L), 0.14(R)	fusiform	0.17(R)
parahippocampal	0.14(L)	inferiortemporal	0.21(R)
rostralanteriorcingulate	0.15(L), 0.16(R)	middletemporal	0.23(R)
rostralmiddlefrontal	0.15(L)	postcentral	0.18(R)

*Supplementary Table S7: Clinical relevance of the deviations across the whole brain; Significant correlation (Spearman) between extreme value across all the regions and symptoms measured by ADOS and ADI scores (P_value < 0.05). * indicates the regions survived after FDR correction*

	**ADI**			**ADOS**		
	ADI-social	***ADI-communication***	***ADI-RRB***	***ADOS-TOTAL***	***ADOS-SA***	***ADOS-RRB***
Female	0.03	0.13	0.16	-0.02	-0.13	0.30*
Male	0.11	0.10	0.20*	0.09	0.03	0.20*

Supplementary References

1. Simonoff E, Pickles A, Charman T, Chandler S, Loucas T, Baird G (2008): Psychiatric Disorders in Children With Autism Spectrum Disorders: Prevalence, Comorbidity, and Associated Factors in a Population-Derived Sample. *J Am Acad Child Adolesc Psychiatry*. 47: 921–929.

2. Wong AYS, Hsia Y, Chan EW, Murphy DGM, Simonoff E, Buitelaar JK, Wong ICK (2014): The Variation of Psychopharmacological Prescription Rates for People With Autism Spectrum Disorder (ASD) in 30 Countries. *Autism Res*. . doi: 10.1002/aur.1391.

3. Loth E, Charman T, Mason L, Tillmann J, Jones EJH, Wooldridge C, *et al.* (2017): The EU-AIMS Longitudinal European Autism Project (LEAP): design and methodologies to identify and validate stratification biomarkers for autism spectrum disorders. *Mol Autism*. 8: 24.

4. Rasmussen CE, Williams CKI (2006): Model Selection and Adaptation of Hyperparameters. *Gaussian Process Mach Learn (Adaptive Comput Mach Learn Ser*. 105–128.

5. Rasmussen CE, Williams CKI (2006): *Gaussian Processes for Machine Learning. Adapt Comput Mach Learn*. . doi: 10.1142/S0129065704001899.

6. Marquand AF, Wolfers T, Mennes M, Buitelaar J, Beckmann CF (2016): Beyond Lumping and Splitting: A Review of Computational Approaches for Stratifying Psychiatric Disorders. *Biol Psychiatry Cogn Neurosci Neuroimaging*. 1: 433–447.

7. Dale AM, Fischl B, Sereno MI (1999): Cortical surface-based analysis: I. Segmentation and surface reconstruction. *Neuroimage*. . doi: 10.1006/nimg.1998.0395.

8. Rosen AFG, Roalf DR, Ruparel K, Blake J, Seelaus K, Villa LP, *et al.* (2018): Quantitative assessment of structural image quality. *Neuroimage*. . doi: 10.1016/j.neuroimage.2017.12.059.

9. Charman T, Loth E, Tillmann J, Crawley D, Wooldridge C, Goyard D, *et al.* (2017): The EU-AIMS Longitudinal European Autism Project (LEAP): clinical characterisation. *Mol Autism*. 8: 27.

10. Goodman R, Ford T, Richards H, Gatward R, Meltzer H (2000): The Development and Well-Being Assessment: description and initial validation of an integrated assessment of child and adolescent psychopathology. *J Child Psychol Psychiatry*. . doi: 10.1111/j.1469-7610.2000.tb02345.x.
